# Cognitive, Affective, and Motivational Changes during Ostracism: An ERP, EMG, and EEG Study Using a Computerized Cyberball Task

**DOI:** 10.1155/2013/304674

**Published:** 2013-11-07

**Authors:** Taishi Kawamoto, Hiroshi Nittono, Mitsuhiro Ura

**Affiliations:** ^1^Graduate School of Integrated Arts and Sciences, Hiroshima University, 1-7-1 Kagamiyama, Higashihiroshima 739-8521, Japan; ^2^Japan Society for the Promotion of Science, 5-3-1 Kojimachi, Chiyoda 102-0083, Japan; ^3^Department of Psychology, Otemon Gakuin University, 2-1-15 Nishiai, Ibaraki 567-8502, Japan

## Abstract

Individuals are known to be highly sensitive to signs of ostracism, such as being ignored or excluded; however, the cognitive, affective, and motivational processes underlying ostracism have remained unclear. We investigated temporal changes in these psychological states resulting from being ostracized by a computer. Using event-related brain potentials (ERPs), the facial electromyogram (EMG), and electroencephalogram (EEG), we focused on the P3b amplitude, corrugator supercilii activity, and frontal EEG asymmetry, which reflect attention directed at stimuli, negative affect, and approach/withdrawal motivation, respectively. Results of the P3b and corrugator supercilii activity replicated findings of previous studies on being ostracized by humans. The mean amplitude of the P3b wave decreased, and facial EMG activity increased over time. In addition, frontal EEG asymmetry changed from relative left frontal activation, suggestive of approach motivation, to relative right frontal activation, indicative of withdrawal motivation. These findings suggest that ostracism by a computer-generated opponent is an aversive experience that in time changes the psychological status of ostracized people, similar to ostracism by human. Our findings also imply that frontal EEG asymmetry is a useful index for investigating ostracism. Results of this study suggest that ostracism has well developed neurobiological foundations.

## 1. Introduction

People are sensitive to social exclusion. Sometimes, a slight social rejection evokes emotional pain in excluded individuals [1–7]. According to an evolutionary perspective, sensitivity to social exclusion is necessary for human survival [4, 8], because social isolation is often a fatal threat [9, 10]. Therefore, humans have developed monitoring systems that are highly sensitive to cues indicative of social exclusion [11, 12].

It is known that people can detect the slightest cue indicative of ostracism and as a result develop aversive feelings. Laboratory studies have demonstrated that ostracism during a computerized ball-tossing game called Cyberball triggers negative affect and lowers the degree to which individuals are able to fulfill their four fundamental needs: belonging, control, self-esteem, and meaningful existence [4]. This effect was observed even when participants knew that the ostracizing others were computer-generated players or when they knew beforehand that they would be ostracized [7]. Functional magnetic resonance imaging (fMRI) studies have shown that social exclusion provokes social pain, which is reflected in activation of the dorsal anterior cingulate cortex (dACC) [1]. Since this region is also associated with physical pain [13, 14], it has been proposed that social and physical pain share a common neural alarm system [15]. These findings suggest that people are sensitive to being accepted or excluded by others, and that their emotional reactivity to these social situations can be mapped onto specific neural circuitry. 

Despite extensive research on ostracism, relatively few studies have focused on the time course of changes in psychological states during such situations. Instead, prior research has focused primarily on postexclusion feelings or experiences of overall social exclusion. It is important to understand the temporal dynamics of psychological states underlying ostracism, because reactions to ostracism are considered to be dynamic processes [16], such that ostracism is associated with specific emotional states and cognitive efforts to reappraise the situation [1, 4]. In their pioneering work, Themanson et al. demonstrated that attention to exclusionary cues decreased with time [17]. These results were also corroborated by an fMRI study [18]. In addition, a behavioral study using a self-report measure has revealed that negative affect in response to ostracism increased with time [19]. Other studies have investigated neural and psychophysiological mechanisms that are closely related to appraisal and regulation of aversive impact of ostracism [20–22]. However, it is unclear whether temporal changes in psychological status also occur in response to ostracism by a computer-generated opponent. In addition, relatively few studies have included multiple psychophysiological measures in one study design. In the present study, we used physiological measurements and investigated the time course of cognitive, affective, and motivational processes that are associated with ostracism by computer-generated opponents.

It is known that social exclusion is one of the causes of psychological problems including depression [23] and aggression, as has been reported in cases of school shootings [24]. Therefore, it is important to understand ostracism in more detail, in order to develop countermeasures against the psychological difficulties that it causes. It is known that using a computer-generated opponent can enhance experimental validity, because computers are the perfect research confederates [25]. Thus, it is valuable for future research to clarify whether ostracism by a computer or a computer-generated opponent can also cause aversive feelings and psychological changes similar to ostracism by humans. Based on the studies suggesting that people respond to humans and computers in a similar manner [25, 26] and that ostracism from computer-generated opponents evokes social pain [3], we predicted that ostracism by computer-generated opponents would evoke subjective and neurobiological responses similar to ostracism by human. 

To investigate cognitive processes, we measured event-related potentials (ERPs). Among the various ERP indices, we focused on the P3b component, because this component has been extensively studied in relation to cognitive processing under unpredictable situations and also because it has been the focus of social exclusion studies. The P3b component has been associated with multiple cognitive functions, including attentional allocation and task-relevance evaluation [27]. This component is elicited by a task-relevant event and shows a larger amplitude for a more significant event [28, 29] or for events with a lower subjective probability [30, 31]. The amplitude of the P3b component is generally interpreted as an index of attention directed at eliciting stimuli [30, 32, 33]. A previous study has indicated that P3b amplitude in response to exclusionary cues is associated with self-reported need threat and that this amplitude decreased with time [24]. Therefore, we predicted that P3b amplitude would be larger during the initial phase of an interaction and that this amplitude would be associated with self-rated social pain. 

 Facial electromyogram (EMG) activity was utilized to investigate affective processes. Facial EMG activity over the corrugator supercilii, which is located above the eyebrows, is inversely related to the valence of a subjective experience. In general, pleasant stimuli elicit less activity and unpleasant stimuli elicit more activity than neutral stimuli [34]. Since this activity has been a reliable measure of negative affect [35, 36], the negative affect induced by social exclusion should be reflected by increased facial EMG activity over the corrugator supercilii. A previous study has reported that negative affect increased with time [19]. Therefore, we predicted that facial EMG activity would increase with time during social exclusion.

 Electroencephalogram (EEG) was utilized in order to investigate the motivational processes associated with social exclusion. It has been demonstrated that asymmetrical activity in the frontal lobes within the alpha band (i.e., alpha power) is related to motivation and/or emotional factors. Alpha power is inversely related to cortical activation [37]. Previous research has demonstrated that activation of the left frontal cortex is associated with approach-related motivation, whereas that of the right frontal cortex is associated with withdrawal-related motivation [38–41]. In terms of the alpha asymmetry index (i.e., ln[right alpha power] − ln[left alpha power]), a positive score reflects higher right frontal alpha power (i.e., relative left frontal activity) and a negative score reflects higher left frontal alpha power (i.e., relative right frontal activity). Given the previous finding indicating that P3b amplitude and dACC activation in response to exclusion cues decreased with time [17, 18], ostracized people may consider shifting the motivational direction to effectively cope with ostracism. Since participants cannot interact with others during ostracism, we predicted that relative right frontal activity (i.e., withdrawal motivation) would be observed in the later phase of an interaction.

 In the present study, participants were asked to play a simple ball-tossing game called Cyberball [5] with two computer-generated opponents [7]. In addition to the exclusion condition, we set up two comparison conditions: a *fair play* condition, which has been used as a control condition in prior research [1, 2, 5, 7], and an *observation* condition, designed to compare the variation of temporal changes in psychological states across conditions. In the observation condition, participants observed the players tossing the ball to each other. The exclusion condition was similar to the observation condition, with the exception that the participant becomes aware that the ball is not being tossed to him or her. The observation condition was used to control (1) the probability that the ball does not come to the participant and (2) participant's responses when he or she needed to throw the ball to the other players. In the fair play condition, participants joined in the game equally with the other two players. To examine the time course of cognitive, affective, and motivational processes during exclusion, the first and second halves of the observation, exclusion, and fair play conditions were analyzed separately, similar to previous studies [17, 18].

 We tested three hypotheses in the present study. First, P3b amplitude during the exclusion condition would be larger in the initial phase of an interaction (i.e., first half) than in the later phase of an interaction (i.e., second half). Second, corrugator supercilii activity during the exclusion condition would be larger in the later phase of an interaction than in the initial phase of an interaction. Finally, relative right frontal activity (i.e., withdrawal motivation) would be observed in the later phase of an interaction relative to the initial phase of an interaction during the exclusion condition.

## 2. Materials and Method

### 2.1. Participants

Healthy undergraduates (*N* = 19, 11 women and *M*_age_ = 18.3 years) attending Hiroshima University, in Higashi-Hiroshima city, Japan, participated in the experiment. All participants were right handed, as assessed by the Edinburgh Handedness Inventory [42]. Research Ethics Committee of the Graduate School of Integrated Arts and Science, Hiroshima University, approved the research protocol. All participants gave their written informed consent for participation in the study.

### 2.2. Design and Materials

 The original Cyberball task [5] was modified for the ERP to make the timing of the ball toss clearer and more discernible. Participants were instructed to practice their mental visualization skills during the game. They were told that performance in the game was unimportant because the game was merely a means for them to engage their mental visualization skills. Similar to a previous study [7], participants in this study were also aware that other players did not actually exist. 

**Figure 1:**
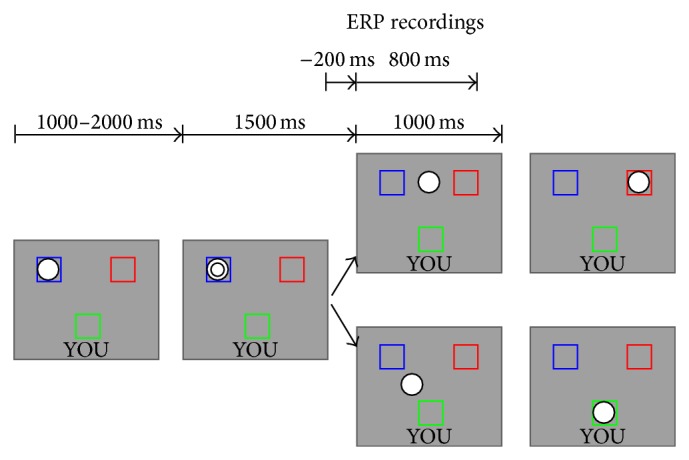
A schematic diagram of the modified Cyberball task used in the present study. The ball (a single circle) was changed to a double circle 1500 ms before tossing. Then, the ball moved to the participant or to the other player. The computer players held the ball for a random period lasting between 1000 and 2000 ms. When the ball appeared, the participant selected to toss it to the left or right player by pressing one of two buttons. ERPs were recorded time locked to the ball toss of the computer players.

Figure 1 shows a schematic diagram of the task. Two computer-generated opponents (blue and red squares) appeared at the top right and the top left corners of the screen. The participant appeared as a green square with the word “YOU” at the bottom of the screen. To predict the timing of the ball toss more precisely, the ball (a single circle) was programmed to change to a double circle 1500 ms before being tossed. The ball traveled for 1000 ms until another player received it. Participants used their left and right index fingers placed on a response pad to toss the ball to the player on their right or left.

 The Cyberball task was used in all three conditions. Although the primary interest was in the last, exclusion condition, the typical procedure used in previous studies (i.e., the order of conditions) was followed [1, 43, 44]. First, participants were asked to observe the other two computer-generated opponents tossing the ball to each other (observation condition), which lasted for 45 trials (approximately 150 seconds). Then, participants joined in the game with the other two computer-generated opponents (fair play condition). This fair play condition consisted of three blocks, each consisting of 45 trials (approximately 150 seconds) as to control the length of the time across the condition. During fair play, computer-generated opponents threw to each other 45 times (not receiving the ball) and threw to the participant 45 times (inclusion). The participant threw back to the computer-generated opponents the remaining 45 times. After these two conditions, the participants unknowingly completed a block in which the other computer-generated opponents suddenly excluded the participant (exclusion condition). In this block, the ball was tossed to the participant only once on the first trial and was not tossed to the participant during the subsequent 45 trials (approximately 150 seconds).

 During short breaks between the blocks, participants completed a questionnaire that assessed social pain [7, 45–47]. Social pain was measured using four statements designed to assess the participants' subjective experiences (e.g., “I felt liked,” “I felt rejected,” “I felt invisible,” and “I felt powerful”). These experiences were rated on a 9-point scale ranging from 1 (Not At All) and 9 (Very Much). Two items, “I felt liked” and “I felt powerful,” were reverse scored such that higher scores for each item indicated a greater level of social pain. The average value of the four items was used as a social pain index. 

### 2.3. Physiological Measurement, Recordings, and Processing

The EEG was recorded at 39 scalp sites (Fp1/2, AFz, Fz, F3/4, F7/8, FCz, FC1/2, FC5/6, FT9/10, Cz, C3/4, T7/8, CPz, CP1/2, CP5/6, TP9/10, Pz, P3/4, P7/8, POz, PO9/10, Oz, O1/2, and Iz) using Ag/AgCl electrodes on an elastic cap (EASYCAP GmbH, Germany). Vertical and horizontal electrooculograms were recorded from electrodes attached above and below the left eye and at the outer canthi. Electrode impedances were less than 5 KΩ. The signal was recorded with a bandpass filter of 0.016–120 Hz at a sampling rate of 500 Hz using a digital EEG amplifier (Nihon-Kohden, EEG1100). The data were rereferenced to the linked earlobes offline. A digital bandpass filter of 0.1–60 Hz and 0.1–100 Hz was applied for the ERP and EEG, respectively. Ocular artifacts were corrected using the method of Gratton et al. [48] implemented in Brain Vision Analyzer 1.05 (Brain Products, Germany). In our design, the fair play condition differed from the other two conditions with regard to participants' response (i.e., throw to other players) and direction of the ball (i.e., receiving the ball from other players). To prelude the influence of these differences, ERP results were analyzed only for trials in which participants did not receive the ball during observation, fair play, and exclusion conditions. ERP waveforms were obtained by averaging a 1000 ms period between 200 ms before and 800 ms after the stimulus onset (i.e., the period of the ball movement) separately for the first half (i.e., the first 20 trials where the ball did not come to the participant) of the block for observation and exclusion conditions. Because fair play was conducted for three blocks, the first half contains the first 7 trials where the ball did not come to the participant across blocks (i.e., 21 trials) and the second half contains the last 7 trials where the ball did not come to the participant across blocks (i.e., 21 trials). After visual inspection, trials that contained nonocular artifacts were excluded from averaging. Artifact-free trials were then averaged (*M* = 18.66, SD = 1.90, range: 12 to 21 trials). The amplitude of P3b was measured at Pz as the mean amplitude that was observed 350 to 450 ms after stimulus onset, based on the ranges used in previous studies [17, 22] and visual inspection of the grand mean waveforms in the present study.

 An EMG over the corrugator supercilii was recorded using a pair of miniature electrodes above the left eye, according to the recommendation of Fridlund and Cacioppo [49]. Electrode impedances were less than 5 KΩ. The sampling rate was 500 Hz. The raw EMG signal was filtered with a high-pass filter of 90 Hz and rectified [50]. Each block was subdivided into 15 temporal segments (10 sec each: *T*_1_–*T*_15_) and the mean EMG amplitude in each segment was calculated. Any segment with an amplitude value exceeding the mean ± 3 standard deviations of each participant was regarded as an outlier and linearly interpolated using the values of the former and subsequent segments (this involved less than 5% of the segments). Then, the change of EMG activity from the beginning (*T*_1_) was calculated (variation index: Log *T*_*n*_ − Log *T*_1_, where *n* was segment number (1 to 15)). To investigate temporal changes similar to ERP, the mean EMG activity was calculated separately for the first half (i.e., average from *T*_2_ to *T*_8_) and for the second half (i.e., average from *T*_9_ to *T*_15_) of the block. Because fair play was conducted in three blocks, average activity (i.e., first and second halves) of the three blocks was used as EMG activity during fair play. To check the difference across fair play blocks, we conducted a 3 (block: first fair play block versus second fair play block versus last fair play block) × 2 (segment: first half versus second half) ANOVA, but there was no statistically significant main effect of block and interaction between block and segment (*F*(1,18) = 0.46, *P* = 0.66, *F*(2,36) = 1.37, *P* = 0.27, resp.).

Alpha power from lateral frontal sites (*F*7, *F*8) was calculated by fast Fourier transform using a Hamming window within the alpha band (8–13 Hz) separately for the first half (i.e., the first 70 seconds) and the second half (i.e., the last 70 seconds). Asymmetry scores were then calculated by subtracting the natural log of alpha power from the left electrode (*F*7) from the corresponding electrode over the right hemisphere (*F*8). Because fair play was conducted in three blocks, average activity (i.e., first and second halves) of the three blocks was used as an asymmetric index during fair play. To assess the difference across fair play blocks, we conducted a 3 (block: first fair play block versus second fair play block versus last fair play block) × 2 (segment: first half versus second half) ANOVA, but there was no statistically significant main effect of block and interaction between block and segment (*F*(1,18) = 1.68, *P* = 0.21, *F*(2,36) = 1.28, *P* = 0.29, resp.). Since alpha power is inversely related to cortical activity, higher alpha power on the left side relative to the right side indicates greater activity in the right than the left [37].

## 3. Results

### 3.1. Subjective Ratings

**Figure 2:**
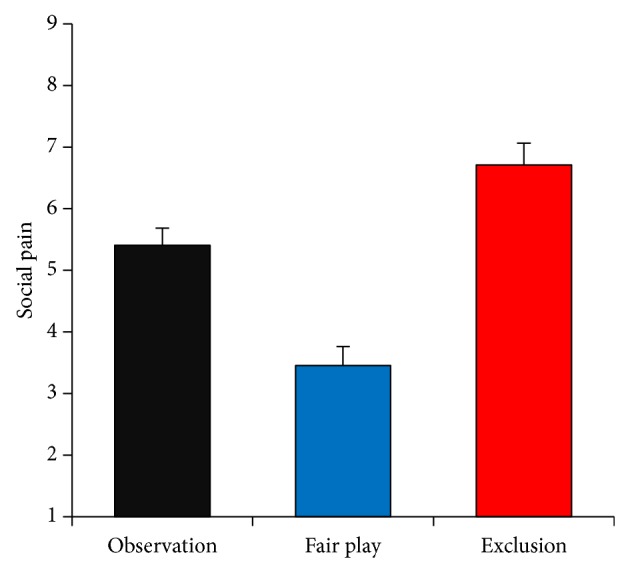
Subjective social pain scores for observation, fair play, and exclusion conditions. Higher scores indicate higher social pain. Error bars indicate the standard error of means across participants.

As seen in Figure 2, the mean social pain ratings were 5.41, 3.46, and 6.71 for the observation, fair play, and exclusion conditions, respectively. To check the effectiveness of the manipulation, a one-way repeated measures analysis of variance (ANOVA) was conducted on the social pain ratings. There was a significant main effect of condition (*F*(2, 36) = 57.37, *P* < 0.05, *η*_*p*_^2^ = 0.76). Post hoc comparisons using paired *t*-tests with Bonferroni corrections showed that these means all differed significantly from each other (*P*s < 0.05). These results confirmed that social pain was effectively elicited during the exclusion condition. 

### 3.2. ERP

**Figure 3:**
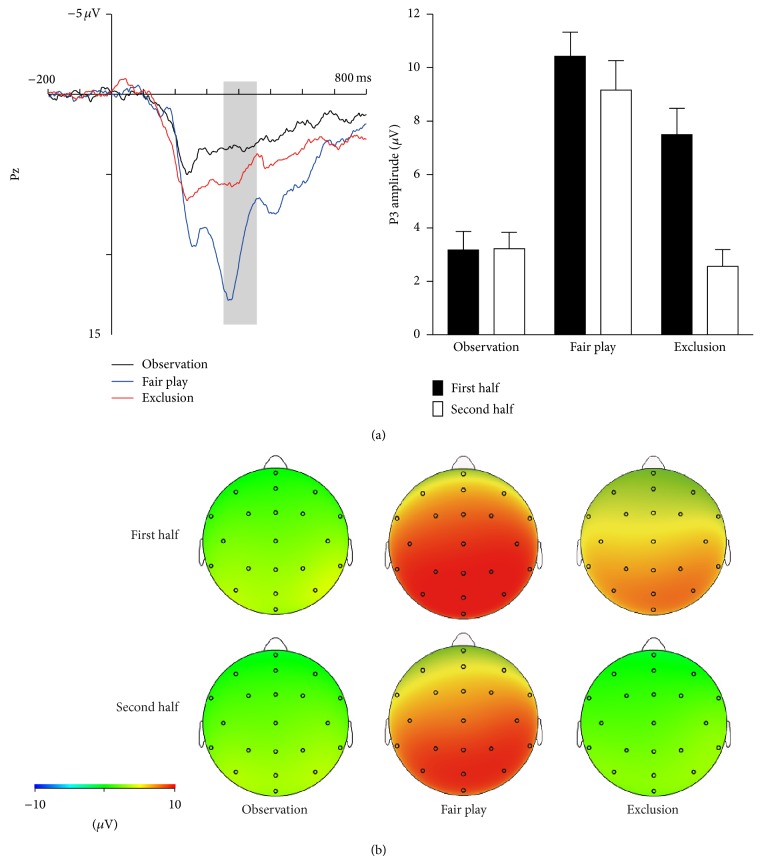
Results of ERP. (a) Grand mean ERP waveforms at Pz elicited by ball tosses to the other player during observation, fair play, and exclusion (left side) and P3b amplitudes (350–450 ms) in the first half and second half of blocks in each condition (right side). Error bars indicate standard errors of means across participants. (b) Topographical map of the first and second half of blocks in each condition.

Figure 3 shows the grand mean ERP waveforms in the observation and exclusion conditions (left side) and the mean P3b amplitudes in the first and second halves of the exclusion and observation conditions at Pz (right side). A 3 (condition: observation versus fair play versus exclusion) × 2 (segment: first half versus second half) ANOVA showed a significant main effect for condition (*F*(2, 36) = 30.04, *P* < 0.05, *η*_*p*_^2^ = 0.63) and segment (*F*(1, 18) = 27.36, *P* < 0.05, *η*_*p*_^2^ = 0.60). The interaction between condition and segment was also statistically significant (*F*(1, 36) = 10.79, *P* < 0.05, *η*_*p*_^2^ = 0.36). Post hoc analysis revealed that P3b amplitude during the exclusion and fair play conditions was larger for the first half block than for the second half block (*P*s < 0.05), although this difference was not statistically significant during the observation condition (*P* = 0.97). In addition, these means for the first and second half blocks were larger during fair play condition than during observation and exclusion conditions (*P*s < 0.05). Furthermore, P3b amplitude for the first half block was larger during exclusion condition than during observation condition (*P* < 0.05), while this difference was not significant in the second half block (n.s.). 

 To examine the changes in P3b amplitude, a one-way repeated ANOVA was performed on the difference value. The difference value was calculated by subtracting P3b amplitude in the first half block from that in the second half block. There was a statistically significant main effect of condition (*F*(2, 36) = 10.79, *P* < 0.05, *η*_*p*_^2^ = 0.36). Post hoc comparisons showed that the difference value was more negative during the exclusion condition than during the observation and fair play conditions (*P*s < 0.05). There was no statistically significant difference between observation and fair play conditions (*P* = 0.61). 

**Figure 4:**
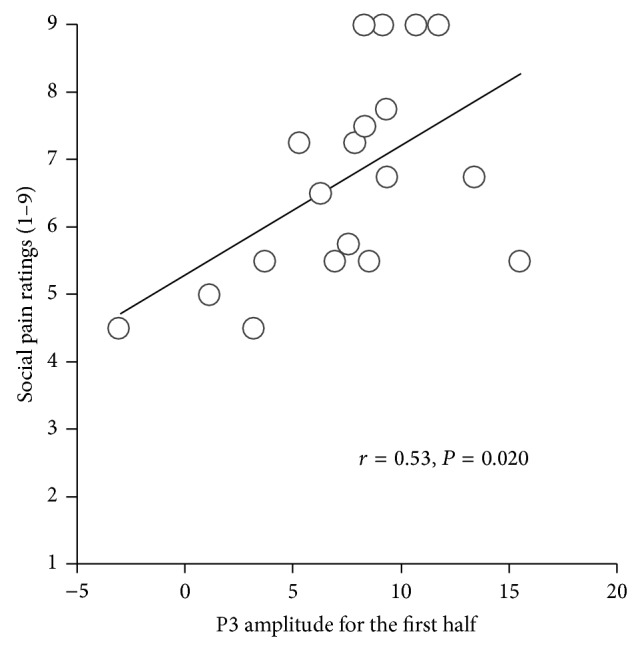
Scatter plots of P3b amplitude and self-rated social pain scores during social exclusion. *X*-axis indicates P3b amplitude for the first half of blocks during social exclusion.

To examine the relationship between P3b amplitude and subjective ratings during exclusion, Pearson's correlation coefficients were computed. P3b amplitude during the first half exclusion block was positively correlated with social pain (*r*(17) = 0.53, *P* < 0.05; see Figure 4), whereas P3b amplitude during the second half exclusion block was not statistically significantly related to social pain (*r*(17) = 0.15, *P* = 0.40). In addition, there was a weak correlation between the difference value and social pain (*r*(17) = −0.45, *P* = 0.06). However, the relationship between the difference value and social pain was not statistically significant after controlling the P3b amplitude during the first half block (*r*(17) = −0.07, *P* = 0.77).

### 3.3. EMG

**Figure 5:**
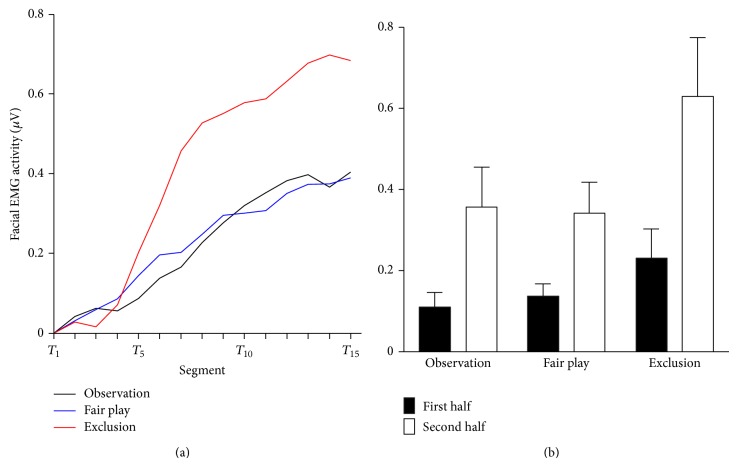
The results of EMG. Time course of EMG activity over the corrugator supercilii during the observation, fair play, and exclusion conditions (a) and the mean EMG activities aggregated in the first half and second half of blocks in each condition (b). Error bars indicate the standard error of means across participants.

Figure 5 shows the mean time course of facial EMG (i.e., corrugator supercilii) activity within a block of each condition (left side) and in the first and second halves of the observation, fair play, and exclusion blocks (right side). A 3 (condition: observation versus fair play versus exclusion) × 2 (segment: first half versus second half) ANOVA showed a statistically significant main effect of condition (*F*(1, 36) = 4.48, *P* < 0.05, *η*_*p*_^2^ = 0.20) and segment (*F*(1, 18) = 16.61, *P* < 0.05, *η*_*p*_^2^ = 0.48). The interaction between condition and segment was also statistically significant (*F*(1, 36) = 6.93, *P* < 0.05, *η*_*p*_^2^ = 0.28). Post hoc analyses revealed that facial EMG activity for the second half block was larger during the exclusion condition than during the observation (*P* < 0.05) and fair play conditions (*P* = 0.07), while these differences were not statistically significant for the first half block (*P*s > 0.25). In addition, corrugator supercilii activity in the second half block was significantly larger than in the first half block for all conditions (*P*s < 0.05). 

To examine the relationship between facial EMG activities and subjective ratings during exclusion, Pearson's correlation coefficients were computed. Facial EMG activity during the first half of the exclusion block was weakly correlated with social pain (*r*(17) = 0.42, *P* = 0.08). In contrast, both facial EMG activities during the second half exclusion block and the difference value were not statistically significantly related to social pain (*r*s < 0.38, *P*s > 0.11). 

### 3.4. Frontal EEG Asymmetry

**Figure 6:**
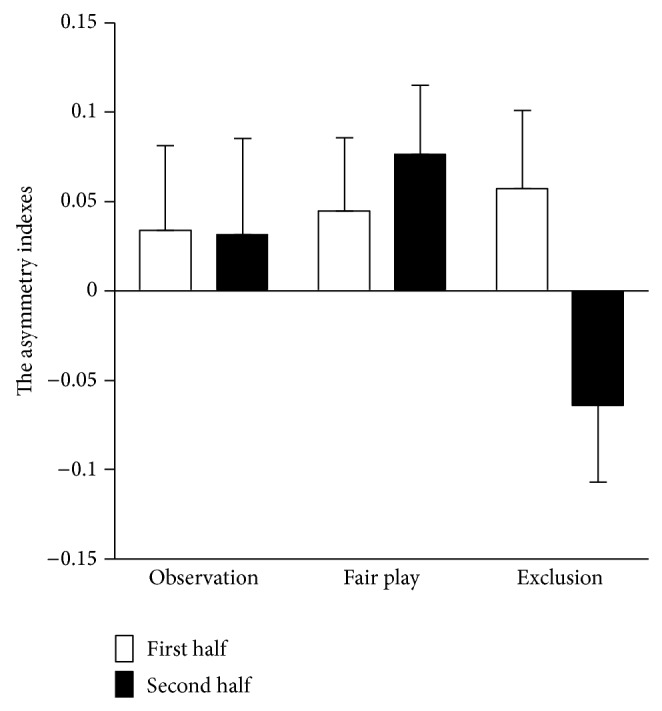
Frontal asymmetry indexes for the first and second halves in each condition. Positive score indicates approach motivation, whereas negative score indicates withdrawal motivation. Error bars indicate the standard error of means across participants.

Figure 6 shows the mean asymmetry scores for the first and second halves of the observation, fair play, and exclusion conditions. A 3 (condition: observation versus fair play versus exclusion) × 2 (segment: first half versus second half) ANOVA showed a marginally significant main effect of condition (*F*(1, 36) = 2.46, *P* = 0.10, *η*_*p*_^2^ = 0.12). The interaction between condition and segment was statistically significant (*F*(1, 36) = 8.04, *P* < 0.05, *η*_*p*_^2^ = 0.31). Post hoc analyses revealed that the asymmetry index for the second half block was more negative during the exclusion than during the observation and fair play conditions (*P*s < 0.05), while these differences were not statistically significant for the first half block (n.s.). In addition, the asymmetry score during the exclusion condition was significantly more negative for the second half block than for the first half block (*P* < 0.05), while this difference was not statistically significant during observation and fair play conditions (*P*s > 0.16). 

To examine the relationship between asymmetry score and subjective ratings during exclusion, Pearson's correlation coefficients were computed. No statistically significant relationships were found. 

## 4. Discussion

We investigated the time course of cognitive (i.e., P3b amplitude), affective (i.e., facial EMG activity), and motivational processes (i.e., frontal EEG asymmetry indexes) while being ostracized by a computer-generated opponent, by using a modification of the Cyberball task. We simultaneously recorded ERPs elicited by exclusion cues, facial EMG activity over the corrugator supercilii, and frontal EEG activities. Consistent with the predictions of the study, P3b amplitude during the exclusion condition was larger in the first half, relative to the second half of the task. In addition, corrugator supercilii activity during social exclusion was larger in the second half than in the first half of the task. Furthermore, during the exclusion condition, we observed relatively stronger left frontal activity (i.e., approach motivation) in the first half of the task, whereas relatively stronger right frontal activity (i.e., withdrawal motivation) was observed in the second half of the task. Such changes were not detected or were only weekly observed in the observation and fair play conditions in which there was no ostracism. These findings suggest that cognitive, affective, and motivational processes resulting from being ostracized by a computer-generated opponent were similar to changes resulting from being ostracized by humans [17–19]. 

Participants felt more social pain during the exclusion condition, as has been reported in previous studies [1, 4]. In other words, participants felt social pain, even without conducting higher-order processing to conduct the excluders' mental state, or intention. This finding implies that social pain can be caused by superficial cues such as the frequency of ball throws. Because ostracism causes various psychological problems [23, 24], people have a detection system that is highly sensitive to ostracism [11, 12]. Our findings strongly suggest that people can detect the slightest cue of ostracism and as a result experience aversive feelings. This idea was also confirmed by the result of the observation condition; although social pain was lower during observation than exclusion, participants felt some social pain in the observation condition compared to the inclusion condition. 

Patterns of physiological measurements and self-rated items identified cognitive and affective processes underlying ostracism. First, attention to exclusion cues decreased with time, as was indicated in a previous study [17]. It is possible that the first experience of being excluded elicits strong aversive responses associated with violated expectations of fair play and detection of social exclusion. These may decline after multiple rejections. In line with this idea, previous research has shown that during social exclusion, dACC activity, which serves to detect social exclusion and for social pain appraisal [1, 47], was larger in the initial phase of an interaction than in the later phase of an interaction [18]. Such findings suggest that once an individual detects social exclusion, he or she moves attention away from exclusion cues. In the present study, the P3b amplitude in the first half of the task during the exclusion condition was strongly and positively correlated with subjective social pain. This finding is consistent with the notion that social pain triggers an individual's attention to focus on the source of an exclusion, so as to determine if the exclusion is potentially threatening or important [4]. Our findings suggest that greater attention to exclusion cues in the initial phase of an interaction increases immediate social pain. 

Interestingly, we also found that P3b amplitude in the second half of the block did not differ between the observation and exclusion conditions; attentional allocation to the exclusionary cues seems to be similar in both conditions at the later stage of the interaction. This finding raises some possible alternative interpretations regarding the results of P3b. The Cyberball task includes multiple factors related to the participants, which change over time. These include subjective probability and personal significance of events and such factors may also affect P3b amplitude [28–33]. Thus, in the later stage of the interaction during ostracism, participants might have noticed that they would never receive the ball. Accordingly, the results of P3b can be interpreted not only by merely ostracism-specific responses, but also task-relevant factors such as self-relevance or stimulus probability. Exactly which factors—subjective social pain or stimulus probability—reflect the processing of ostracism is a matter of debate [15, 47, 51]. Ostracism is a complicated phenomenon that inherently involves lower subjective probability and self-relevance. Therefore, it is difficult to break ostracism into distinct components. Nevertheless, it would be valuable for future research to investigate whether the decline in the P3b amplitude reflects merely ostracism-specific response or other factors.

Second, results indicated that negative affect increased with time, which was also consistent with a previous study [19]. It should be noted that all conditions tended to increase facial EMG activities over time. This was possibly due to mental fatigue, which was involved in all the conditions [52]. What is more important, however, is that facial EMG activity increased dramatically in the exclusion condition as compared to the other conditions. Findings of this study regarding the P3b suggest that negative affect may be evoked even after attention to exclusion cues has moved away from such cues indicating that negative affect induced by exclusion was not determined only by exclusion cues, but by the exclusion situation as a whole. Although our facial EEG data showed that negative affect increased over time, previous findings suggest that negative affect evoked by ostracism quickly recovers [19, 53]. Thus, it could be the case that negative affect accumulates over time and decreases at a certain point. By taking previous findings and our results into consideration, it would be possible to conclude that there is a time lag between negative affects induced by ostracism and cognitive efforts to regulate these negative affects. However, the issue of whether negative affects recover over time and how they recover remains unknown. It is suggested that future studies may benefit from investigating the recovery process from negative affect during ostracism, by manipulating the length of ostracism and by focusing on individual differences. 

The changes in frontal EEG found in this study are to the best of our knowledge the first evidence of temporal changes in frontal EEG during ostracism. This finding may suggest that the direction of motivation shifted from an approach state to a withdrawal state [38–42] because of ostracism. It is known that ostracism is a deeply aversive event, and therefore, once individuals detect that they are being socially excluded, they may feel, think, and behave so as to reestablish the optimal level of their fundamental need to belong [4]. In ostracism studies using Cyberball, participants do not have an opportunity to interact with others during social exclusion, because the other players do not exist in the same room. In this situation, because participants cannot engage with the simulated other players, they cannot reestablish the optimal levels of fundamental needs through the responses of other players. Our findings imply that the shift in motivation to a withdrawal state during the exclusion condition reflects withdrawal from the situation of social exclusion and/or emotional withdrawal. In support of this idea, findings of this and previous studies have indicated that attention and dACC activation to exclusion cues decrease over time [17, 18]. Since social exclusion is an aversive experience, individuals withdraw from exclusionary situations or screen themselves from the negative effects of social exclusion. In corroborating this possibility, previous studies have indicated that ostracized people can quickly ameliorate the negative effect of ostracism [19, 53], suggesting that people can effectively regulate the negative effects of ostracism. The link between the asymmetry observed during ostracism and withdrawal-related motivation and relationship between withdrawal-related motivation and the processes of ameliorating the effects of ostracism should be examined in future studies. Such investigations would help fill the gap in knowledge resulting from the highly limited number of investigations on ostracism using frontal EEG [22, 54].

Although the observation and exclusion conditions evoked social pain compared to the inclusion condition, the time course of psychophysiological activities was considerably different. This finding is in line with previous studies indicating that implicit social exclusion, such as when people could not participate in a Cyberball task due to an Internet malfunction, also evoked social pain; however that direct regulation of social pain did not occur in these cases [1, 43, 44]. Our findings also provided evidence that an ostracism-like event—the observation condition—did not change psychological status. One possible reason for this could be that people in the observation condition did not need to change their psychological status, because they knew beforehand not that they should join the task. That is, they could easily change the meaning of ostracism before the task. Despite this, an ostracism-like event—the observation condition—still evoked social pain, which supports the notion that even the slightest hint of ostracism evokes social pain [1–7]. More research is needed to investigate the types of ostracism events that cause direct regulation processes and psychological changes, by focusing on the intensity and awareness about ostracism. 

There were several limitations to this study. First, similar to prior research [1, 43, 44], the presentation order of our conditions was always identical (observation, fair play, and exclusion). It is possible that order and sequence effects may have affected our results. Previous studies on ostracism have suggested that mere order effects do not cause the subjective and neurobiological responses, such as social pain or dACC activity, in response to social exclusion [47]. Nevertheless, it is suggested that future studies should address this issue by implementing the task condition as a between-subjects factor or by randomizing the order of the conditions. Second, we measured social pain in short breaks between blocks. It is possible that social pain rating may affect the cover story and participants' perception of ostracism. In some studies measured social pain was given after all experimental conditions were completed [1, 45–47], whereas other studies have measured it immediately after each condition [43, 44]. In addition, the fact that we observed ostracism-related psychological changes and increases in subjective social pain suggests that our design was adequate to produce the phenomena of interest. Nevertheless, future research is required to investigate whether the frequency of social pain rating affects the meaning of ostracism. Third, although neither the exclusion, nor the observation condition involved motor responses, the fair play condition did involve a button-press response to throw the ball. Previous studies have implied that P3b was affected by motor preparation and motor responses [55–57]. Therefore, ERP differences among the conditions might be partly due to movement-related activities. Although the fair play condition (i.e., natural social interactions) inherently involves motor responses, further studies would be needed to control the effects of motor preparation and motor response on ERPs by using other paradigms or stimuli, such as the rejection paradigm [51] and facial expression stimuli [58, 59]. Forth, there are individual differences in response to ostracism. For example, it is known that people that are low in self-esteem have higher self-reported social pain, because they hyperactivate the appraisal process (e.g., dACC) in response to ostracism [46]. On the other hand, people high in general trust are known to effectively regulate social pain [43]. In addition, individual differences and neurobiological responses to ostracism may interact to predict postrejection aggression [60]. Thus, future studies would benefit from investigating how individual differences modulate changes in the psychological status during ostracism. Finally, the present sample size was relatively small. Further studies should rectify this shortcoming by investigating a larger sample to see if the results of our study would be replicated. 

## 5. Conclusion

We have provided evidence that ostracism by computer-generated opponents is an aversive experience and that psychological condition during ostracism changes over time. More specifically we found that as a result of ostracism, attention to exclusion cues decreased with time, whereas negative affect increased with time and motivation shifted to a withdrawal pattern. The present findings suggest that ostracism has well developed neurobiological foundations.
